# Enhanced Silhouette InstaLift™ Technique for Precision Lifting: A Technical Innovation and Retrospective Analysis of Personal Experience in 100 Consecutive Cases

**DOI:** 10.7759/cureus.107943

**Published:** 2026-04-29

**Authors:** Tso-Hsuan Wang, Wan-Ling Tu

**Affiliations:** 1 Plastic Surgery, Nice Clinique, Taipei, TWN

**Keywords:** case mixes, complication, minimally invasive facial lifting, silhouette instalift, thread lift

## Abstract

Background: The Silhouette InstaLift™ (Sinclair Pharma, Irvine, California, United States) has gained popularity as a minimally invasive technique with an effective and safe profile, offering immediate results and minimal downtime compared to barbed suture thread lifting. This study introduces a modified Silhouette InstaLift™ technique designed to enhance precision in thread placement and reduce complications. We report our experience on the clinical outcomes and safety of this modified technique.

Methods: We retrospectively analyzed 100 consecutive cases treated between 2018 and 2023. All procedures were performed by a single surgeon using the modified Silhouette InstaLift™ technique. The approach involved precise thread placement and multi-point fixation to enhance the stability of the lift. Outcomes were assessed using the Global Aesthetic Improvement Scale (GAIS) at postoperative day 1, one month, three months, and six months. Complications were recorded and analyzed.

Results: The mean thread count used was 6.1±2.3. A small proportion of patients (7%) utilized other types of threads, and 3% underwent concurrent procedures. Complications were minimal, with 6% of patients experiencing dimples and 2% reporting unevenness. GAIS assessments showed that 90% of patients reported improvement on post-procedural day 1, which increased to 94% by six months. The trend demonstrated a sustained and even a continuous increase in aesthetic improvement over time.

Conclusions: The modified Silhouette InstaLift™ technique provides enhanced precision in lifting, resulting in improved aesthetic outcomes and a lower complication rate. It can prevent cheekbone protrusion, provides greater stability than the original method, and leads to long-term satisfaction. Further research with larger and more diverse populations is recommended to validate this novel approach and explore long-term outcomes.

## Introduction

The pursuit of effective yet minimally invasive facial rejuvenation techniques has led to the development of various thread-lifting procedures [1-3]. Thread lifting was pioneered by Sulamanidze and Sulamanidze in the 1990s [4]. Initially, conventional sutures yielded suboptimal long-term results. To overcome this limitation, barbed sutures, known as Aptos threads, were developed, significantly enhancing the effectiveness of thread-lifting procedures. Since then, several variations of thread-lifting techniques have emerged, including ptosis correction, Isse endo Progressive Facelift Sutures, Silhouette Sutures, and Contour Threads. These innovations have become increasingly popular among aesthetic practitioners [5-9].

Among these, the Silhouette InstaLift™ (Sinclair Pharma, Irvine, California, United States) has emerged as a notable non-surgical alternative to traditional facelifts [10,11]. Launched in 2015, Silhouette InstaLift™ features absorbable sutures with bi-directional cones, engineered to lift and reposition midfacial tissues while promoting collagen production for enhanced long-term results. Silhouette InstaLift™ sutures are composed of 82% poly-L-lactic acid (PLLA) and 18% poly(glycolide/lactide) (PLGA). These sutures are equipped with bi-directional cones that assist in lifting and repositioning tissue while also stimulating collagen production for sustained results. This technique addresses the increasing demand for procedures that provide immediate improvement with minimal downtime, making it particularly appealing to patients in their 40s to 60s who prefer less invasive alternatives to traditional surgery [10-12].

While the original Silhouette InstaLift™ has been widely recognized for its effectiveness, it is not without challenges, as is the case with all aesthetic procedures. Commonly reported complications include swelling, bruising, infection, asymmetry, and skin dimpling similar to those observed with other types of threads, such as polydioxanone (PDO) barbed threads [12-14]. Additionally, it may result in cheekbone protrusion, a particular concern among the Asian population.

In response to these challenges, we developed a modified Silhouette InstaLift™ technique aimed at enhancing the precision of thread placement and reducing the risk of undesirable outcomes. Specifically, this approach is designed to optimize the lifting of the nasolabial and jawline fat compartments to achieve a more natural contour while minimizing the risk of over-correction or distortion. The objective of this study was to describe this modified technique and to evaluate its clinical performance and safety profile in a retrospective case series. Outcomes were assessed using the Global Aesthetic Improvement Scale (GAIS) and procedure-related complications across multiple postoperative time points. Importantly, this study was not designed to establish superiority over existing methods.

## Materials and methods

Study design

This study was designed as a retrospective case series.

Patient recruitment

Consecutive patients treated at our clinic (Nice Clinique, Taipei, Taiwan) between 2018 and 2023 were included. Patient data were collected through a retrospective chart review. All procedures were performed by a single surgeon (Dr. Wang) using the modified Silhouette InstaLift™ technique described below.

Inclusion and exclusion criteria

The inclusion criteria were consecutive patients who underwent the modified Silhouette InstaLift™ procedure. The exclusion criteria were patients with incomplete clinical records or insufficient follow-up data for outcome evaluation.

Data collection

Clinical data were retrospectively reviewed from available medical records. Procedural characteristics, including the number of threads used and technical modifications applied, were recorded.

Outcomes

Outcomes were evaluated using the GAIS, which categorizes results as follows: 5 indicates "very much improved", 4 represents "much improved", 3 signifies "improved", 2 corresponds to "no change", and 1 denotes "worse" [15,16]. This scale measures the surgeon's assessment of the aesthetic results. GAIS assessments were performed by the treating surgeon based on standardized clinical photographs obtained at each follow-up visit.

Ethics statement

Formal institutional review board (IRB) approval was waived due to the retrospective nature of the study. Written informed consent for the procedure and use of anonymized clinical photographs was obtained from all patients.

Statistical analysis

Descriptive statistics were used to summarize patient characteristics and GAIS outcomes. Continuous variables were presented as mean±standard deviation or median (interquartile range), and categorical variables were presented as number (percentage). Given the descriptive nature of the study, no formal hypothesis testing was performed.

Surgical technique

The modified Silhouette InstaLift™ technique introduced in this study represents a significant advancement over the original Silhouette InstaLift™ technique [4], with a focus on the precise determination of the lifting distance and pre-establishing exit points. The technique also emphasizes securing knots without applying downward pressure and incorporates a secondary fixation on the superficial temporal fascia to enhance and achieve a highly stable outcome. The superficial temporal fascia was selected as the anchoring layer due to its accessibility and lower risk of injury to deeper neurovascular structures, although a comparative biomechanical evaluation was not performed in this study. These improvements not only reduce the risk of adverse effects, such as cheekbone protrusion, but also ensure a more stable and lasting lifting effect. All procedures were performed under standardized clinical conditions at a single center. The following section provides a detailed description of the surgical technique employed in this innovative approach.

Thread Selection, Positioning, and Design

The procedure begins with careful identification of the target fat layers, primarily focusing on the nasolabial fat of the midface and the jawline regions. Each targeted lifting area is treated with a minimum of two threads to ensure sufficient structural support. In general, when both the upper and lower facial regions are addressed simultaneously, four threads are required on each side. However, the number of threads may be reduced in cases where the fat layer is continuous. The procedure utilized an eight-cone thread in more than 90% cases. In cases involving patients with an elongated facial structure or requiring an extended lifting distance, a 12-cone thread was selected.

Sectioning and Lifting Direction

**Figure 1:**
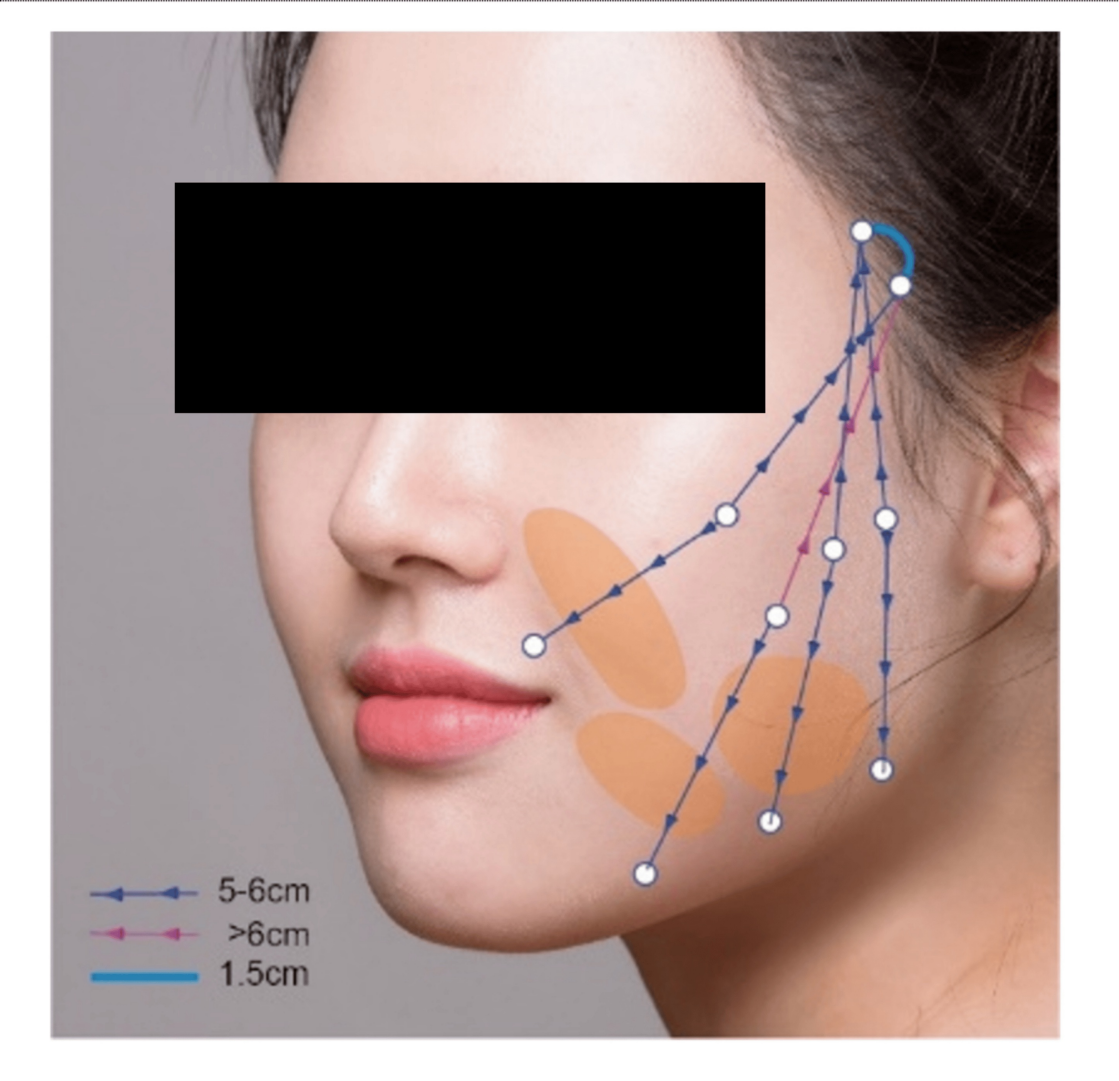
Schematic illustration of the modified Silhouette InstaLift™ technique demonstrating the planned entry and exit points and the knotting locations for a four-thread placement pattern Illustration created by the authors using licensed stock images (Shutterstock, Shutterstock, Inc., Empire State Building, New York City, New York, United States) and finalized using Adobe Illustrator (Version 27.9, Adobe Inc., San Jose, California, United States).

To reposition the posterior fat layer of the jawline effectively, the fat layer is divided into three equal parts. Two low points within these sections are designated as the primary lift points. The lifting distance is precisely calibrated between 5 and 6 cm, with meticulous planning of the lifting direction to avoid any interaction with the cheekbones. This careful approach ensures that the lifting does not extend beyond the cheekbone area, thereby preventing any unwanted cheekbone protrusion. If the lifting distance reaches 6 cm, it may interfere with the cheekbone (i.e., the zygoma), so we have set the distance at 5.5 cm or lower as a precaution. After determining the optimal lifting direction at the second point, the third point is prioritized based on the shortest required distance, maintaining the same 5-6 cm range. Figure 1 is the schematic illustration shows the details for four-thread placement (Figure 1).

Incision and Thread Placement

Small incisions are made with an 18G needle at the predetermined entry and exit points using standard surgical instruments. The threads are then carefully inserted into the fat layers through these incisions, ensuring they pass uniformly through the targeted fat layers, with the cones providing secure anchorage. This approach is consistent with the original method, where the threads are positioned within the fat layer and each insertion is meticulously checked to ensure even placement. The technique prioritizes precise thread positioning, which is essential for achieving the desired lifting effect without distorting the natural facial contours.

**Figure 2:**
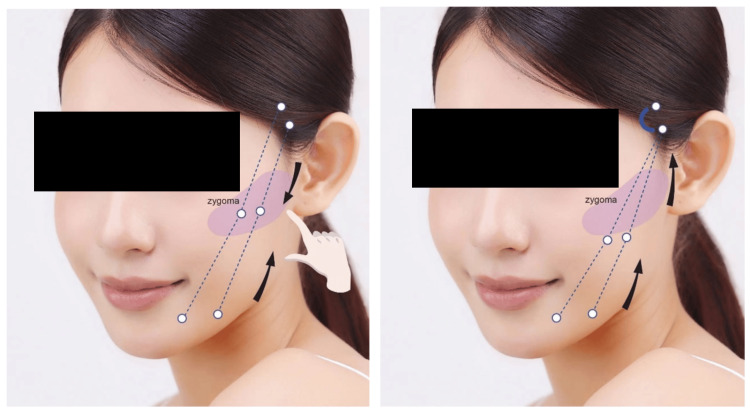
Comparison of the original and modified Silhouette InstaLift™ tensioning strategy. The original technique (left) involves both upward and downward tensioning of the threads, whereas the modified technique (right) applies upward tensioning only. The modified vector design aims to reduce the risk of cheekbone (zygomatic) protrusion and to achieve more balanced facial contours Illustration created by the authors using licensed stock images (Shutterstock, Shutterstock, Inc., Empire State Building, New York City, New York, United States) and finalized using Adobe Illustrator (Version 27.9, Adobe Inc., San Jose, California, United States).

Our modified approach involves carefully planning and creating exit points to guide the needle out in a more controlled manner, unlike the original Silhouette InstaLift™ method where the needle can exit wherever it reaches within the fat layer. Additionally, while the original method involves both upward and downward tensioning of the threads, our modified technique employs only upward tensioning. This adjustment is made to prevent cheekbone protrusion, ensuring balanced and natural facial contours. Figure 2 illustrates the thread entry and exit points, along with the path and tensioning directions.

Fixation and Knotting

**Figure 3:**
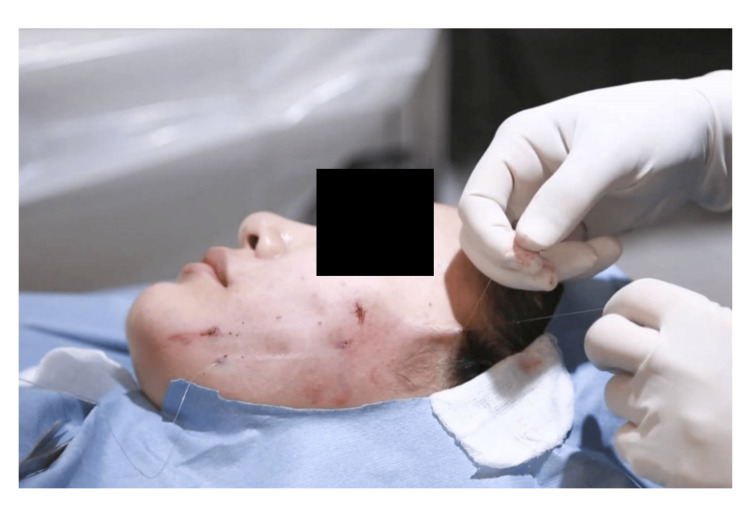
Intraoperative photograph demonstrating the fixation step of the modified technique. Two threads exit through the same point at the edge of the hairline and are subsequently tied to secure fixation

The threads are fixed at carefully selected points along the hairline, specifically at its edge rather than within the scalp, to facilitate easier knot tying. Typically, the fixation point is located in the middle of the temporal area. After all the threads are placed through the same incision, they are tied off with knots for stability (Figure 3). The second thread is usually longer than 6 cm to accommodate easier knot tying. This knotting process further elevates and securely anchors the structure, preventing downward movement and distinguishing this technique from the original method.

Secondary Fixation

To further enhance the stability and longevity of the lift, a secondary incision is made 1.5 cm from the original knotting point. This additional incision serves as an exit point for threading and knotting, ensuring that the threads are securely anchored through the superficial temporal fascia. After tying the initial knot, the threads are carefully passed through this secondary point and firmly knotted. A C-shaped hook (also called a U-shaped guided needle) (see Appendices) is used to facilitate this process.

The rationale for this approach is that, although the knotting site serves as an anchor point, it is essentially a floating anchor since the entire thread is positioned within the fat layer. To reinforce the fixation, a secondary anchoring step is performed, securing the thread to the superficial temporal fascia. This step is crucial for maintaining the lift over an extended period, particularly in areas prone to gravitational forces.

The effectiveness of the fixation can be verified by observing the movement of the head when the C-shaped hook is pulled. If the fascia has been properly engaged, the entire head will move with the pull; if only the fat layer is engaged, the head will remain stationary. Finally, three to four additional knots are tied to achieve maximum stability. This secondary fixation not only enhances the immediate lifting effect but also prolongs the duration of the results.

Completion and Inspection

Upon completing the threading and knotting, any excess thread material is carefully trimmed at the skin's surface. The incisions are then closed, and the treated areas are thoroughly inspected to ensure that the threads are evenly distributed and the lift is symmetrical. The final outcome is a well-supported facial structure with enhanced contouring in the nasolabial and jawline regions, free from common side effects such as cheekbone protrusion.

Four-Thread Cases

In cases requiring four threads, two threads are first exited through the first incision point, while the other two threads are exited through the second incision point. The threads from the first incision are tied together, and the same is done for the threads from the second incision. Then, the threads from the first incision are brought up and exited through the second incision point, resulting in four threads at that point. These four threads are then paired and tied together, which greatly enhances the stability of the lift.

Three-Thread and Two-Thread Cases

In some cases, due to clients' budget constraints or when a less extensive lift is required, the procedure may be designed using three threads. For enhanced stability, two threads are passed through the lower exit point and one through the upper exit point. The two lower threads are tied first and then threaded through the upper point using a C-shaped hook and knotted together with the upper thread to complete the fixation.

When patients require lifting in specific areas, such as the nasolabial folds or jawline, only two threads may be used. In these cases, an exit point is made 11-12 cm from the target area. After securing the first knot, the threads are passed through the second incision point, located 1.5 cm above the first, and tied again to achieve secondary fixation.

**Figure 4:**
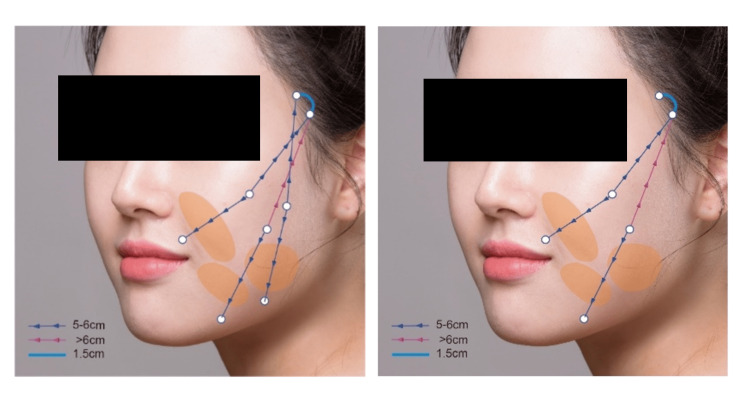
Schematic illustration showing the entry and exit points, thread paths, and knotting locations for alternative thread configurations. The left panel illustrates a three-thread placement, and the right panel illustrates a two-thread placement Illustration created by the authors using licensed stock images (Shutterstock, Shutterstock, Inc., Empire State Building, New York City, New York, United States) and finalized using Adobe Illustrator (Version 27.9, Adobe Inc., San Jose, California, United States).

Figure 4 shows the schematic illustration of the detailed entry and exit points, the paths of the treads, as well as knotting locations, for the cases of three- and two-thread placements.

Postoperative Care

Postoperative management followed routine clinical practice at our institution. Specifically, following the procedure, the wounds are treated with tissue glue (SurgiSeal ® adhesive, H.B. Fuller Medical Adhesive Technologies, LLC, St. Paul, Minnesota, United States). Patients are provided with standard postoperative care instructions, which include avoiding high-impact activities and excessive facial movements for a specified period. Follow-up visits are scheduled to monitor the healing process and ensure that the threads remain securely in place.

All patient photographs were anonymized by obscuring identifying facial features.

## Results

A total of 100 patients were included. The mean age was 48.5±10.0 years, and 92% were female. The mean thread count was 6.1±2.3 (range: 2-14). Additional thread types were used in 7% of cases, and 3% of patients concurrently underwent other procedures.

**Figure 5:**
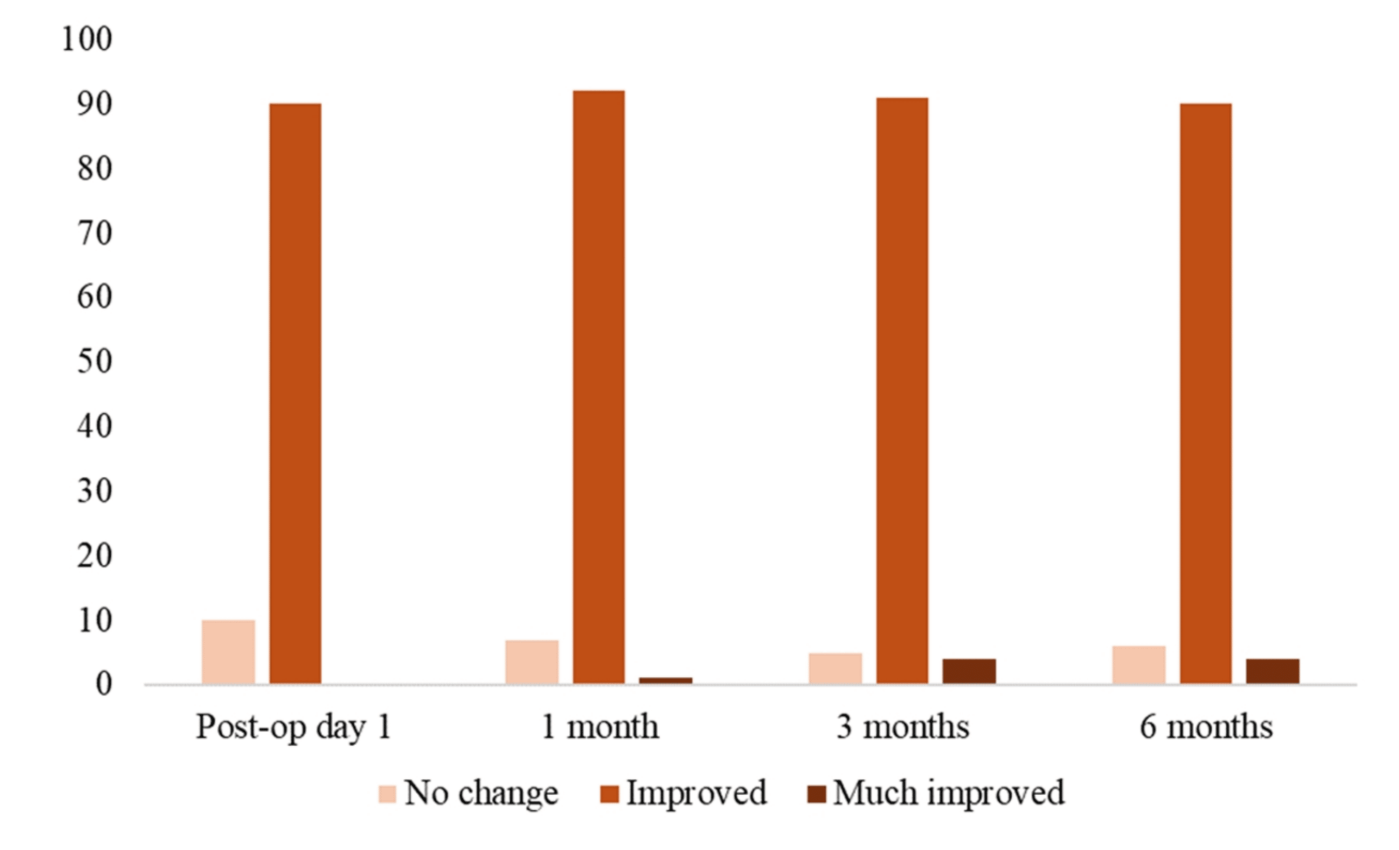
Distribution of GAIS outcomes at postoperative day 1, one month, three months, and six months following the modified Silhouette InstaLift™ procedure GAIS: Global Aesthetic Improvement Scale

Aesthetic outcomes were evaluated using GAIS at multiple postoperative time points. On postoperative day 1, 90% of patients were rated as improved and 10% as no change. At one month, 93% demonstrated improvement (92% improved and 1% much improved), with 7% showing no change. At three months, 95% demonstrated improvement (91% improved and 4% much improved), and at six months, 94% remained improved (90% improved and 4% much improved). Overall, GAIS outcomes indicated sustained aesthetic improvement over time, with a gradual increase in the proportion of "much improved" ratings during follow-up (Figure 5).

**Figure 6:**
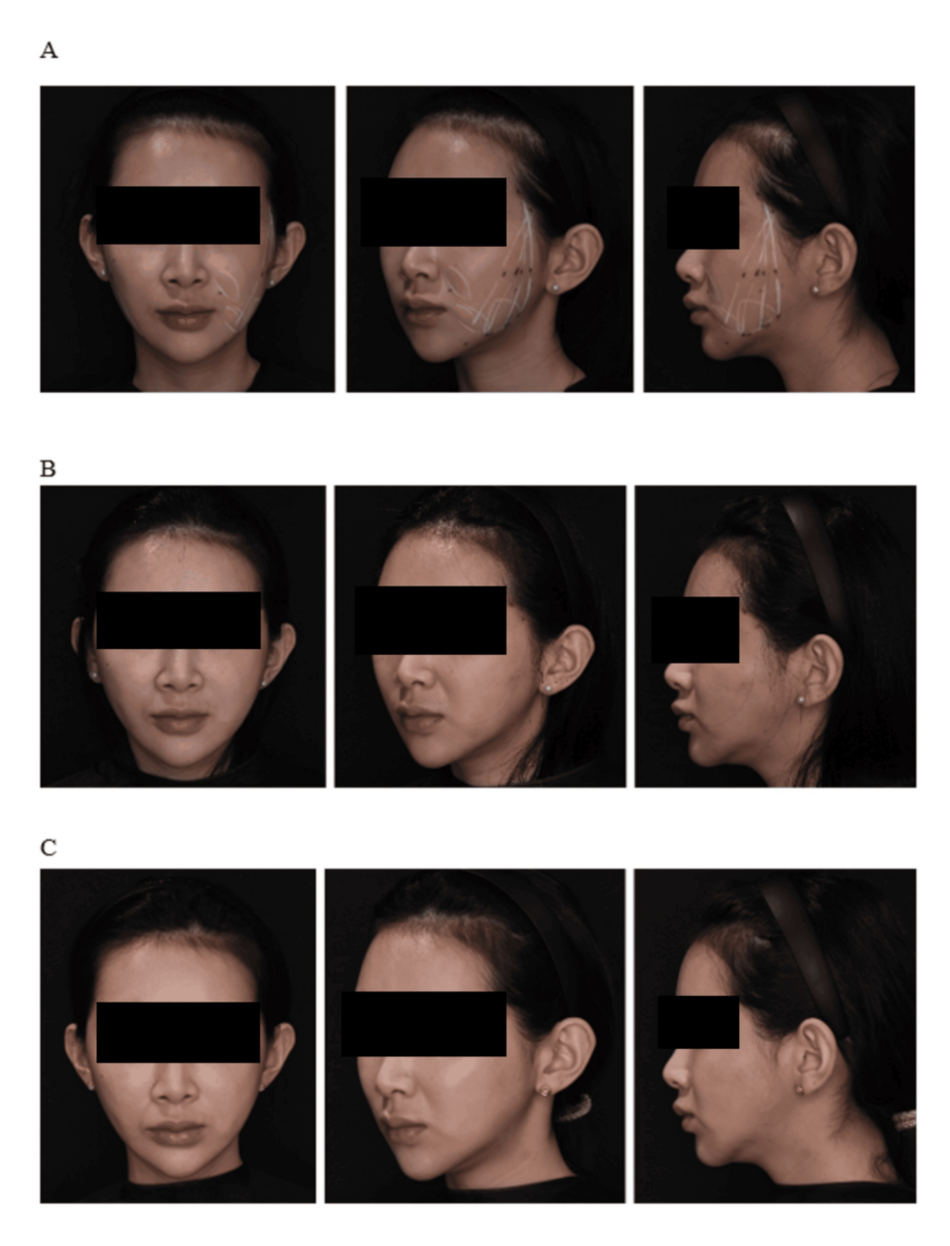
Representative clinical photographs of Case 1 (33 years old) before and after the modified Silhouette InstaLift™ procedure: (A) before the procedure, (B) five days after the procedure, and (C) one month after the procedure

**Figure 7:**
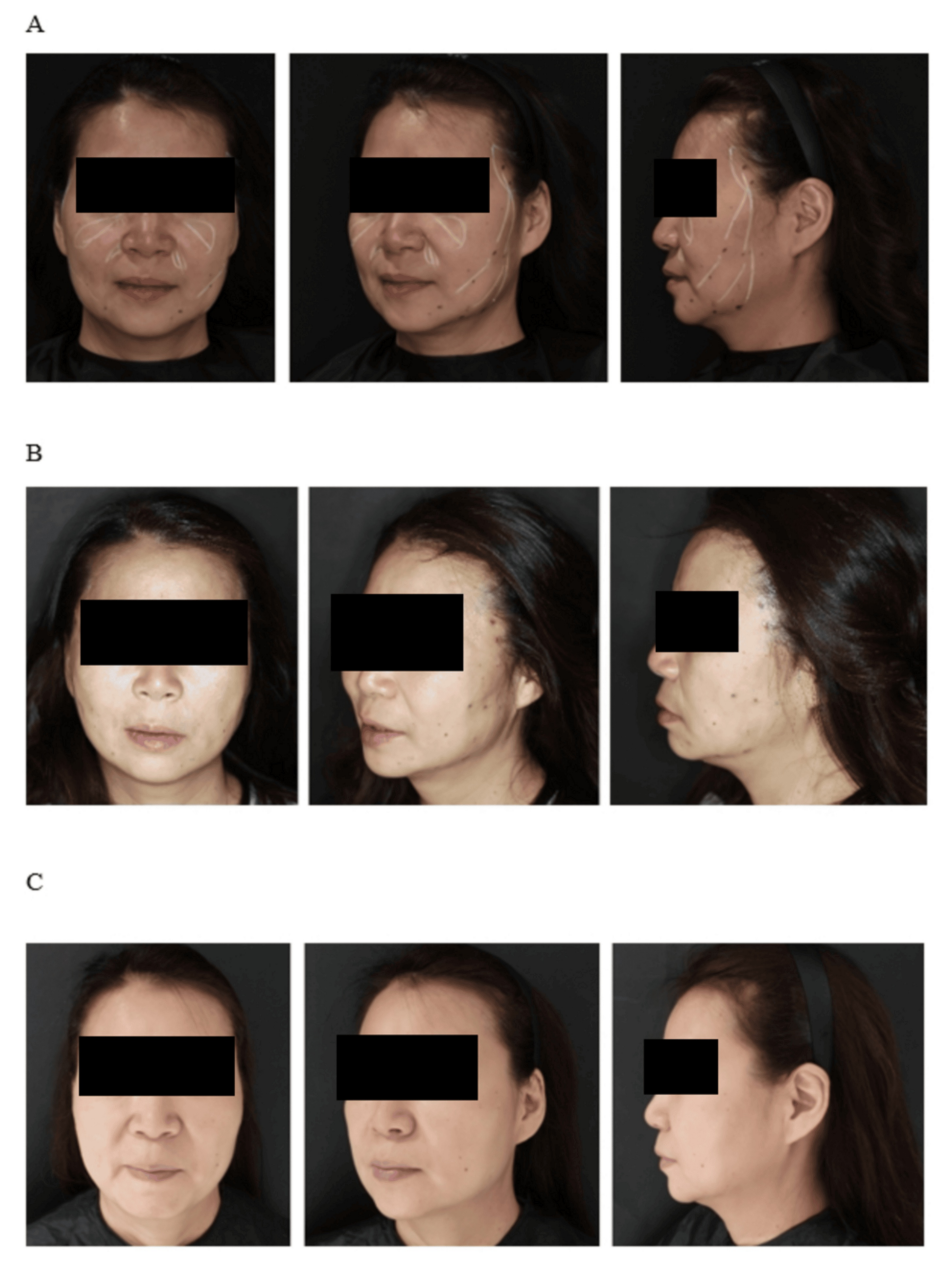
Representative clinical photographs of Case 2 (55 years old) before and after the modified Silhouette InstaLift™ procedure: (A) before the procedure, (B) one day after the procedure, and (C) six months after the procedure

**Figure 8:**
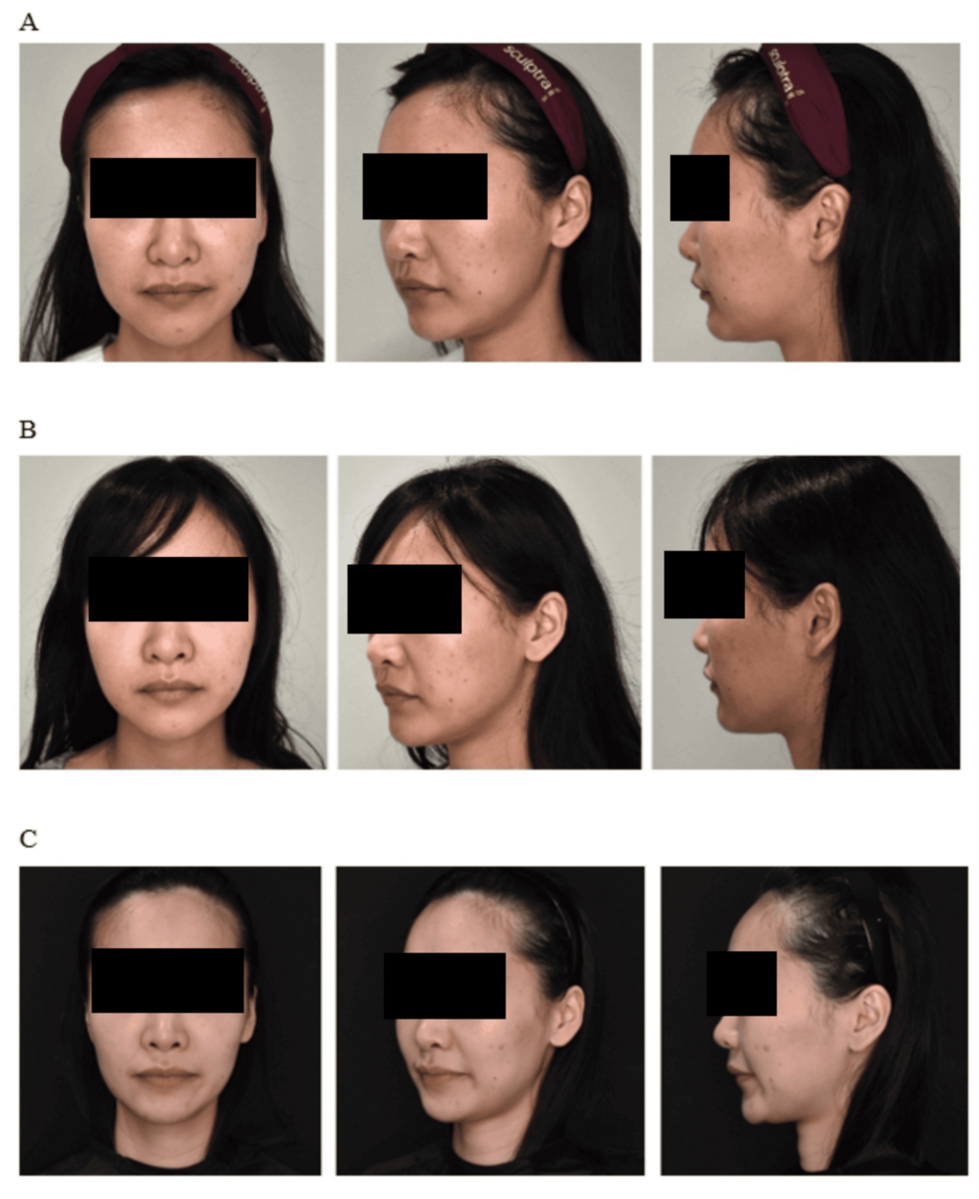
Representative clinical photographs of Case 3 (32 years old) before and after the modified Silhouette InstaLift™ procedure: (A) before the procedure, (B) one week after the procedure, and (C) 1.5 months after the procedure

Representative before-and-after photographs of three cases are shown in Figures 6-8, demonstrating the postoperative aesthetic changes achieved with the modified Silhouette InstaLift™ technique at different follow-up time points.

Safety outcomes

Overall, the modified Silhouette InstaLift™ procedure demonstrated a favorable safety profile. Minor complications were infrequent, with dimpling observed in 6% of patients and asymmetry in 2%. Importantly, no cases of cheekbone (zygomatic) protrusion were reported during the follow-up period (Table 1).

**Table 1:** Characteristics and outcomes of the cases GAIS: Global Aesthetic Improvement Scale

	Overall (N=100)
Age, years	48.5±10.0 (21-70)
Sex	
Male	8 (8%)
Female	92 (92%)
Thread count	6.1±2.3 (2-14)
Use other threads	7 (7%)
Concurrently undergoing other procedures	3 (3%)
Complication	
Dimple	6 (6%)
Uneven	2 (2%)
Cheekbone protrusion	0 (0%)
GAIS (post-op day 1)	
No change	10 (10%)
Improved	90 (90%)
Much improved	0 (0%)
GAIS (1 month)	
No change	7 (7%)
Improved	92 (92%)
Much improved	1 (1%)
GAIS (3 months)	
No change	5 (5%)
Improved	91 (91%)
Much improved	4 (4%)
GAIS (6 months)	
No change	6 (6%)
Improved	90 (90%)
Much improved	4 (4%)

## Discussion

The modified Silhouette InstaLift™ technique introduced in this study represents a refinement of minimally invasive facial rejuvenation approaches. By incorporating innovative steps such as precise distance determination, pre-establishing exit points, and secondary fixation, this technique effectively addresses common complications associated with traditional thread lifting methods and the original Silhouette InstaLift™ technique, including asymmetry and unnatural facial contours.

Our results demonstrate a clear trend of increasing aesthetic improvement over time, as evidenced by the GAIS assessments at various postoperative intervals. Majority of patients (90%) reported improvement by the first postoperative day, with this figure rising to 94% by six months. This consistent enhancement in outcomes suggests that our modified technique not only meets the immediate cosmetic expectations but also provides durable results. Importantly, this study was not designed as a comparative analysis, and no definite conclusions regarding superiority over conventional techniques should be drawn.

Our observation of sustained aesthetic improvement over time is consistent with prior reports that absorbable suspension sutures can produce progressive and durable contour changes beyond the immediate postoperative period, likely reflecting both mechanical repositioning and tissue response during follow-up. In a prospective, masked, controlled study with extension follow-up, Nestor reported maintained improvement and patient satisfaction up to 12 months after treatment with absorbable suspension sutures, supporting the concept that outcomes may persist and evolve over time [17].

Technical planning has been emphasized as a key determinant of outcomes with Silhouette InstaLift™. Lorenc et al. described the importance of vector planning for optimal tissue repositioning and aesthetic results, highlighting that placement strategy and directional design are central to achieving balanced contouring [10]. Our modification builds upon these principles by standardizing the lifting distance and pre-establishing exit points to improve precision and reduce unwanted contour changes.

With respect to safety, prior reviews of thread-lifting procedures have identified dimpling/irregularity and asymmetry among the most common adverse events, along with less frequent events such as extrusion, infection, and hematoma. Our series demonstrated low rates of dimpling and asymmetry and did not observe cheekbone (zygomatic) protrusion, suggesting that the modifications aimed at controlled vector design and fixation may help mitigate contour-related complications that are frequently discussed in the literature [18].

One of the key technical goals of this modified approach is to minimize the risk of cheekbone (zygomatic) protrusion [10,18]. Through careful determination of the lifting distance and the selection of appropriate exit and fixation points, the procedure ensures that the lifting effect is concentrated where it is most needed, without interacting with the cheekbones. This precision is crucial for maintaining a harmonious facial structure, which is a key concern for patients seeking minimally invasive cosmetic procedures [10].

Another important advantage of this modified Silhouette InstaLift™ technique is the incorporation of a secondary fixation step. This additional fixation, performed on the superficial temporal fascia, significantly enhances the stability and longevity of the lift. By providing a secure anchor point, the secondary fixation ensures that the threads remain firmly in place, even in areas subject to gravitational forces. However, these proposed biomechanical advantages are based on surgical rationale and clinical observation rather than direct quantitative measurement. This approach not only improves the immediate lifting effect but also prolongs the durability of the results, reducing the likelihood of complications such as thread migration or loss of tension over time [10]. The secondary fixation is particularly beneficial in maintaining a more stable and long-lasting facial contour, which is a key factor in patient satisfaction with minimally invasive cosmetic procedures [17].

The low complication rate observed in this study further supports the safety of the modified technique. Dimpling occurred in 6% of patients, and mild asymmetry was observed in 2%, which is consistent with the minor adverse events commonly reported after thread-lifting procedures [18]. Importantly, no cases of cheekbone (zygomatic) protrusion were observed. This reduction in complications can be attributed to the meticulous surgical technique, which emphasizes the accurate positioning and secure fixation of the threads, as well as the multi-point fixation strategy that enhances the stability of the lift over time.

However, several limitations of this study should be acknowledged. First, outcome assessment relied on surgeon-reported GAIS scores, which are inherently subjective and may introduce evaluation bias. Second, the follow-up duration was limited to six months and may not fully reflect the long-term durability of the procedure. Third, this was a single-surgeon, single-center study with a relatively small sample size, which may limit the generalizability of the findings.

In addition, the observational nature of this retrospective case series limits the ability to draw causal inferences regarding the effectiveness of the modified technique. Future studies involving larger and more diverse populations, multiple surgeons, objective outcome measures, blinded assessments, and longer follow-up are warranted. Furthermore, detailed baseline characteristics such as skin type, degree of ptosis, and prior procedures were not fully captured, which may affect the applicability of the findings. Finally, although the modified approach appears promising, optimal patient outcomes may require individualized treatment planning, including adjunctive minimally invasive procedures [19].

## Conclusions

The modified Silhouette InstaLift™ technique represents a feasible and reproducible approach that may provide favorable aesthetic outcomes with a low complication profile. Further comparative and long-term studies are needed to validate its effectiveness. By delivering a more precise and stable lifting effect with fewer complications, this modified technique may offer a potential option for patients seeking minimally invasive facial rejuvenation. Continued research and longer-term follow-up studies are essential to further evaluate the durability and safety of this approach.

## Figures and Tables

**Figure 9:**
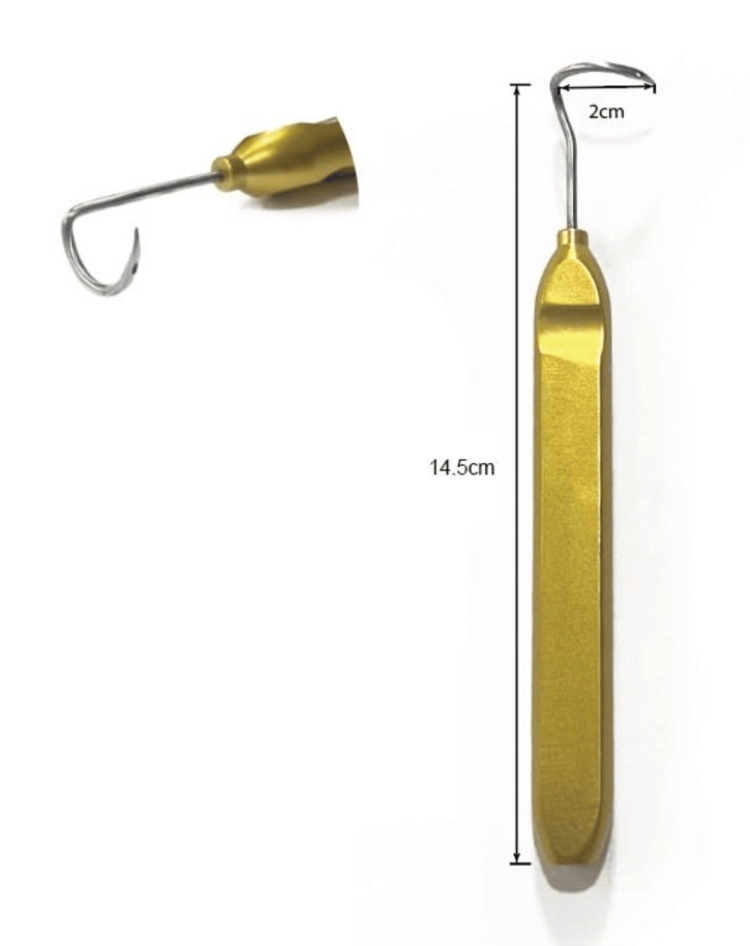
Photograph of the C-shaped hook (U-shaped guided needle) used during the secondary fixation step of the modified Silhouette InstaLift™ technique

## References

[1] Liu Y, Mao R, Xiao M, Zhu W, Liu Y, Xiao H (2024). Facial rejuvenation: a global trend of dermatological procedures in the last decade. Plast Reconstr Surg Glob Open.

[2] Hong GW, Park SY, Yi KH (2024). Revolutionizing thread lifting: evolution and techniques in facial rejuvenation. J Cosmet Dermatol.

[3] Atiyeh BS, Chahine F, Ghanem OA (2021). Percutaneous thread lift facial rejuvenation: literature review and evidence-based analysis. Aesthetic Plast Surg.

[4] Archer KA, Garcia RE (2019). Silhouette InstaLift: benefits to a facial plastic surgery practice. Facial Plast Surg Clin North Am.

[5] Sasaki GH, Cohen AT (2002). Meloplication of the malar fat pads by percutaneous cable-suture technique for midface rejuvenation: outcome study (392 cases, 6 years' experience). Plast Reconstr Surg.

[6] Sulamanidze M, Sulamanidze G (2008). Facial lifting with Aptos methods. J Cutan Aesthet Surg.

[7] Fukaya M (2017). Long-term effect of the insoluble thread-lifting technique. Clin Cosmet Investig Dermatol.

[8] Villa MT, White LE, Alam M, Yoo SS, Walton RL (2008). Barbed sutures: a review of the literature. Plast Reconstr Surg.

[9] Park SY, Kim SB, Suwanchinda A, Yi KH (2024). Non-surgical rhinoplasty through minimal invasive nose thread procedures: adverse effects and prevention methods. Skin Res Technol.

[10] Lorenc ZP, Goldberg D, Nestor M (2018). Straight-line vector planning for optimal results with Silhouette InstaLift in minimally invasive tissue repositioning for facial rejuvenation. J Drugs Dermatol.

[11] Halepas S, Chen XJ, Ferneini EM (2020). Thread-lift sutures: anatomy, technique, and review of current literature. J Oral Maxillofac Surg.

[12] Li YL, Li ZH, Chen XY, Xing WS, Hu JT (2021). Facial thread lifting complications in China: analysis and treatment. Plast Reconstr Surg Glob Open.

[13] Ahn SK, Choi HJ (2019). Complication after PDO threads lift. J Craniofac Surg.

[14] Kochhar A, Kumar P, Karimi K (2022). Minimally invasive techniques for facial rejuvenation utilizing polydioxanone threads. Facial Plast Surg Clin North Am.

[15] Savoia A, Accardo C, Vannini F, Di Pasquale B, Baldi A (2014). Outcomes in thread lift for facial rejuvenation: a study performed with Happy Lift™ revitalizing. Dermatol Ther (Heidelb).

[16] Prantl L, Brix E, Kempa S (2021). Facial rejuvenation with concentrated lipograft-a 12 month follow-up study. Cells.

[17] Nestor MS (2019). Facial lift and patient satisfaction following treatment with absorbable suspension sutures: 12-month data from a prospective, masked, controlled clinical study. J Clin Aesthet Dermatol.

[18] Wang CK (2020). Complications of thread lift about skin dimpling and thread extrusion. Dermatol Ther.

[19] Moon H, Fundaro SP, Goh CL, Hau KC, Paz-Lao P, Salti G (2021). A review on the combined use of soft tissue filler, suspension threads, and botulinum toxin for facial rejuvenation. J Cutan Aesthet Surg.

